# Relationship between Employee Mental Health and Job Performance: Mediation Role of Innovative Behavior and Work Engagement

**DOI:** 10.3390/ijerph19116599

**Published:** 2022-05-28

**Authors:** Xifeng Lu, Haijing Yu, Biaoan Shan

**Affiliations:** 1 College of Accounting, Jilin University of Finance and Economics, Changchun 130117, China; luxifeng621@163.com; 2 School of Business and Management, Jilin University, Changchun 130012, China; shanbiaoan@jlu.edu.cn

**Keywords:** mental health, job performance, innovative behavior, work engagement, emerging economy

## Abstract

The relationship between employee mental health and job performance has been one of the key concerns in workplace. However, extant studies suffer from incomplete results due to their focus on developed economies’ contexts and the unclear path of employee mental health’s impact on performance. In this paper, we investigate the mechanism of employee mental health influencing job performance. We use the data of Chinese firms to test these hypotheses. Drawing on a sample of 239 firms from China, we find that employee mental health positively impacts job performance, and such relationship is mediated by innovative behavior and work engagement. The findings not only enrich the discipline’s knowledge on mental health in an emerging economy setting but also extend the implications of mental health, innovative behavior, and work engagement to job performance.

## 1. Introduction

Employee mental health has long been a topic of concern for researchers and practitioners alike [1]. One reason for this interest is that employee mental health is increasingly prominent within workplaces, which leads to significant costs including absenteeism, burnout, employee compensation claims, work–family conflict and low productivity [2,3]. In particular, with the outbreak of COVID-19, the uncertainties and fears associated with the virus outbreak, along with survival crisis of enterprises, lead to increases in employees’ mental disorders [4,5,6]. For example, Xiong et al. [7] found that people in China, Spain, Italy and five other countries had higher levels of symptoms of anxiety, depression, traumatic stress disorder and other mental health problems during the COVID-19 pandemic. Bufquin et al. [8] showed that since the outbreak, employees in the restaurant industry experienced higher levels of psychological distress and drug and alcohol use than furloughed employees. In this regard, it is timely to examine the influence of the mental health of employees on outcomes.

Recent studies have shown the relationship between employee mental health and different organizational outcomes, including employee emotional expression, job satisfaction, daily work behavior, job performance and firm performance [6,9,10,11,12]. Among these, the relationship between employee mental health and job performance has been an important research topic and has received more and more attention. Scholars suggested that employees with good mental health will show a positive working state and devote themselves to work tasks with more enthusiasm [13], whereas poor mental health may lead to inactivity at work and degradations in interpersonal relationships, which, in turn, negatively impacts employees’ work performance [14,15,16,17].

Although the relationship between mental health and job performance has been well-documented, there still remain some insufficiencies in the previous research. As a result, our extant knowledge on how employee mental health shapes job performance has remained fragmented and limited. First, the path of how employee mental health affects job performance is still unclear. The psychological characteristic–behavior–outcome framework indicates that although a strong individual attribute is important for an outcome, it does not automatically yield that outcome; instead, it influences outcome via appropriate behaviors. Second, such studies have been primarily conducted in Western economic contexts, whereas examinations in Eastern cultures such as China are lacking, which impedes upon the field’s global relevance. Studies have shown that culture, such as individualism and collectivism, will affect individuals’ mental health [18,19]. Therefore, the impact of mental health on performance may be different under different cultural backgrounds.

With the goal of addressing this gap of the unclear path of employee mental health-job performance in the literature, we consider the mediating role of employee innovation behavior and work engagement on this relationship. It additionally aims to identify the antecedents of job performance. Because research on the employee mental health–job performance relationship in emerging economies is lacking, we also aim to analyze the role of employee mental health in job performance in China. To achieve the goals adopted in the study, we examine the employee mental health–job performance relationship, innovation behavior, the work engagement–job performance relationship, and the mediating role of innovation behavior and work engagement in the mental health–job performance relationship using data from Chinese firms. Our results show that employee mental health is positively associated with job performance, and that these effects are mediated by employee innovative behavior and work engagement.

The present paper contributes to the literature in several ways. First, complementing previous research investigating the role of employee mental health in job performance in developed economies, we examine employee mental health’s impact on job performance in China. Second, we contribute to the research on job performance by analyzing the role of innovative behavior and work engagement in job performance. Although prior research has provided insightful understanding of the drivers of job performance, knowledge on job performance could benefit from identifying further drivers that explain this important concept. Third, we explored the pathways of employee mental health affecting job performance ignored by extant research, thus extending the relationship between employee mental health and job performance.

The rest of the article is structured as follows. Firstly, the literature on employee mental health is reviewed, and the corresponding theoretical hypotheses are put forward. Second, this study proposes the methods and results. Then, we conclude by discussing our results and their implications for theory and practice and suggesting future research directions. Finally, we draw a conclusion of this study.

## 2. Literature Review and Hypothesis Development

### 2.1. Employee Mental Health

The World Health Organization [20] defines mental health as “a state of well-being in which the individual realizes his or her own abilities, can cope with the normal stresses of life, can work productively and fruitfully, and is able to make a contribution to his or her community”. Over the years, researchers have developed a variety of operational definitions. For example, Ford et al. [13] suggest that mental health refers to an individual’s affective experiences and behavior. Montano et al. [16] define mental health as a continuum of neurophysiological and cognitive states related to thinking, mood and emotion, and behavior including negative and positive mental health states. Sharma et al. [21] show that mental health is a positive expression, which is the absence of anxiety, social dysfunction and the presence of condition. Based on these definitions, scholars have developed a variety of measurement instruments that include both positive and negative terms in order to describe mental health more accurately [22]. Although definitions and measurements differ among scholars, it is widely accepted that positive affective states are often described as ‘good’ mental health, while a state of emotional suffering such as depression and anxiety is often used to refer to ‘poor’ mental health [8,23].

### 2.2. Employee Mental Health and Job Performance

The relationships between mental health and job performance have received increased attention in the organizational literature. We propose that employee mental health is positively correlated with job performance. This view is consistent with the happy–productive worker hypothesis that suggests that mental health is positively related to job performance [24,25]. Specifically, mentally healthy employees with positive affective states can improve cognitive flexibility and find more solutions to problems in work tasks [26]. Thus, employees with good mental health perform better on work tasks than those with poor mental health. Moreover, positive affective states are associated with individuals building good interpersonal relationships [27], which enable them to receive help from their leaders and colleagues at work. Studies also showed that good social relationships are an important source of job-related information and knowledge [28]. Finally, many studies also support this hypothesis. For example, in a meta-analytic study from 111 independent samples obtained from a search of the literature, Ford et al. [13] indicated that psychological health was a moderate-to-strong correlate of work performance. Similarly, Zacher, Jimmieson and Winter [25] showed that employees’ mental health had a positive effect on work performance in the sample of 165 employees providing in-home eldercare, as well as one colleague and one family member of each employee. At the same time, several meta-analytical findings indicate that poor mental health such as anxiety, depressive symptoms and job stress has a negative impact on job performance [16]. Hence, we expect:

**Hypothesis** **1.** *Employee mental health is positively related to job performance.*

### 2.3. Work Engagement, Innovative Behavior and Job Performance

Work engagement is defined as an active state of work characterized by vigor, dedication, and absorption [29,30]. Engaged employees are energetic and passionate about their work and are often fully immersed in their work [31,32]. In this regard, by investing their cognitive, emotional and social resources into their work, employees can enhance their responsibilities and emotional connection to work, and put more effort and more time into work, which is conducive to achieving high work performance [33]. At the same time, employees with high work engagement have a strong work identity and expect to achieve good results such as high performance through work [34]. Indeed, several studies have shown that work engagement is positively related to job performance [35,36,37].

We further propose that employee innovative behavior has a positive effect on job performance. Innovative behavior is a process of going beyond given paradigms and routines and generating new ideas and implementing them through experimentation [38]. In this process, employees can access a broad range of information to generate creative and new ideas, which facilitate a more detailed understanding of existing problems and alternative solutions through experimentation [39,40]. Moreover, experimentation and trial and error in innovation behavior produce a larger and more elaborate pool of knowledge and involve the recombination or creation of resources [41,42]. This again will facilitate learning and develop capacity [42,43], which in turn improve work performance. Hence, we expect:

**Hypothesis** **2a.** *Employee work engagement is positively related to job performance.*

**Hypothesis** **2b.** *Employee innovative behavior is positively related to job performance.*

### 2.4. Work Engagement and Innovative Behavior as Mediators

Finally, we propose that mental health has an effect on work engagement and innovative behavior, which, in turn, is positively related to work performance. In other words, we argue that work engagement and innovative behavior mediate the relationship between mental health and work performance. Specifically, according to the broaden-and-build theory of positive emotions, positive emotions expand people’s thought–action repertories and build their enduring personal resources including self-efficacy and resilience [26,44]. Studies have shown that such personal resources have a strong motivational potential and are vital antecedents of work engagement [45,46,47,48]. In addition, positive mental health leads to higher work motivation [13]. Employees with positive affect will set high goals for work and expect that engaging in work generates positive outcomes [49]. Finally, positive affect also leads to a heuristic and global information processing pattern that allows employees to concentrate on an ongoing activity, which is an important aspect of work engagement. In contrast, poor mental health such as depression and anxiety is associated with overestimations of risk and underestimations of self-worth, which may lead to lower effort when working [13].

At the same time, the broaden-and-build theory of positive emotions also indicates that positive emotions broaden the array of individual’s existing cognitive frameworks [26], which increases individual cognitive flexibility and the cognitive resources available for recognizing the potential connections between things [44]; this, in turn, helps individuals generate novel ideas not previously available [27,50]. Moreover, some theories propose that affect provides information about the world around us [51,52,53]. A positive emotional state signals that everything is going well and the current situation poses no serious threat [27,50,54,55]. These reactions, in turn, encourage employees to engage in active efforts to try novel things such as innovation [54]. In addition, innovative behavior is a multi-stage process from idea generation to implementation of new and useful ideas within an organization [56,57,58], which is filled with high uncertainty and risks [42,59]. Employees with good mental health have confidence to overcome obstacles in innovation processes and persist longer in efforts to develop and implement innovative ideas [60,61].

In sum, we suggest that the better one’s mental health, the higher their work engagement and innovative behavior. Moreover, work engagement and innovative behavior are shown to be direct antecedents of work performance. Hence, we expect:

**Hypothesis** **3a.** *Work engagement mediates the relationship between mental health and job performance*.

**Hypothesis** **3b.** *Innovative behavior mediates the relationship between mental health and job performance.*

## 3. Methodology

### 3.1. Data Collection and Samples Characteristics

Considering the impacts of COVID-19, an online survey was conducted to test the hypotheses in this study. In order to let participants fully understand the purpose of this survey, we provided important information about the online questionnaire on the first page. In addition, we provided an informed consent at the front of the questionnaire. Additionally, all subjects were required to give their informed consent for inclusion before they started the survey. The questionnaires were from employees’ self-reports. Participants were anonymous and they could quit at any time during the survey. Therefore, this study did not collect any data without consent.

According to the study of Shan et al. [62], the process of developing the questionnaire is shown as follows: First, several employees from different companies were interviewed. This step let us understand more about the core concepts of this study, including job performance and employees’ mental health. Then, we developed a draft questionnaire based on the interviews and previous studies. Finally, we conducted a pilot study to test the preliminary questionnaire. Based on a small-scale survey, we revised the questionnaire and a formal questionnaire for this study was generated. The process could ensure the accuracy of the questionnaire.

Based on the above, this study carried out a formal survey in China. The survey was conducted from October to December, 2021. Participants came from different companies in several provinces, such as Jilin Province, Shandong Province, and Yunnan Province. These companies were located in different regions in China. Our team members sent the questionnaires or links by WeChat, email and other ways. Participants were employees in different positions, such as managers (e.g., middle-level managers) and technical engineers (e.g., employees in production line). About 500 questionnaires or links were sent out to the target employees. We confirmed the samples and eliminated the samples for which the completion rate was low (less than 75%).

Finally, we collected 239 valid samples. These samples came from different industries, including manufacturing industry, construction industry, and service industries. Additionally, the samples were distributed in different regions, such as Northeast, East coast and Southwest China. The characteristics of the samples are as follows. Most participants (73%) are grass-roots employees, and 27% of participants are middle-level or above. 69.2% participants have received a Bachelor’s degree, Master’s degree, or Ph.D., while 30.8% of participants’ education level is low, including Junior college, High school or below. Most participants (66.7%) have worked for less than eight years. Most participants (62.3%) are from mature companies (created more than 10 years ago).

### 3.2. Measurement

We utilized a seven-point Likert scale to measure the core variables in this study. All scales of the related variables have been tested in other studies.

Dependent variable: job performance. Based on the research of Williams and Anderson [63], we utilized four items to measure the variable of job performance. Example items include “I am satisfied with my job performance”, “I could adequately complete assigned duties” and ““I try to work as hard as possible” (Cronbach’s α = 0.858).

Independent variable: Employee mental health. Employee mental health used nine items, mainly from the study of Kashyap and Singh [64]. Example items include “I am always very nervous and feel stressed”, “I always feel unhappy or depressed” and “I always could not concentrate when I do something” (Cronbach’s α = 0.952).

Mediating variables: Work engagement and employee innovative behavior. First, we used four items to measure work engagement based on the study of Suárez-Albanchez et al. [65]. Example items include “I am passionate about my work”, “I feel full of energy when I am working” and “Time flies when I’m working” (Cronbach’s α = 0.849). Second, according to the research of Scott and Bruce [66], we used eight items to measure the variable of employee innovative behavior. Example items include “Always search for ideas for new technologies, processes, and/or products”, “Always generate creative ideas” and “Always promote and support ideas to others” (Cronbach’s α = 0.958).

Control variables: The selection of control variables is important for the results. In this study, firm age, the number of firm employees (firm size), the educational background of employees, and the work experience of employees were set as control variables. Firm age is calculated by the years since the firm was created (1–5 years, 1; 6–10 years, 2; 11–15 years, 3; more than 15 years, 4). Firm size is determined by the number of employees (more than 200 employees, 4; 51–200 employees, 3; 21–50 employees, 2; 20 or fewer employees, 1). Employees’ educational background reflects the levels of employees’ education (high school degree or below, 1; junior college education, 2; Bachelor’s degree, 3; Master’s degree, Ph.D., 4). Work experience refers to the year(s) an employee has worked in the company.

### 3.3. Validity and Common Method Bias

Firstly, we test the possible problem of common method bias. According to a study of Podsakoff and Organ [67], Harman’s one-factor test could be utilized. This method has been widely used in previous studies. The results show that there is no significant problem of common method bias because the largest factor in this study only explained 38.121% of the entire variance. Secondly, the validity of the samples is conducted. The results show that all factor loadings are greater than 0.7 and none were below 0.6. Thus, the validity of the samples is very high in this study.

## 4. Data Analysis and Results

SPSS (IBM, Armonk, NY, USA) was used in this study. First, we calculate the correlations among core variables of this study. The results are shown in Table 1. The coefficients are not high. Second, the descriptive statistics are calculated. We can see that there is no significant problem with the mean and S.D. (standard deviation) of the core variable. All the correlation coefficients do not exceed 0.7. Third, the multicollinearity issue may impact the results. Consequently, we calculate the coefficients of variance inflation factors (VIFs). The results show that there is no VIF that exceeds 10. This indicates that there is no significant multicollinearity based on the view of Hair et al. [68].

Fourth, we apply hierarchical linear analysis (HLA) to test the hypotheses proposed in this study. Seven models are created and the results are shown in Table 2 and Table 3. We control the firm age, the number of firm employees, the educational background of employees, and the work experience of employees. Model 1 showed the results of the impact of three control variables on job performance. The results of model 1 verified that the influence of control variables is not significant. Then, the results of model 2 indicated that H1 was verified. The coefficient for employee mental health is 0.256, which is significant at *p* < 0.01. (Model 2). Therefore, the influence of employee mental health is positive. Model 3 was built to test H2a and H2b. From the results of model 3, both coefficients for work engagement and employee innovative behavior were positive and significant. The results show that work engagement and innovative behavior are positively related to job performance.

In order to test H3a and H3b, we built models 6 and 7 (Table 3). The results show that the impact of employee mental health on work engagement is positive (Model 6: β = 0.284; *p* < 0.001). Additionally, the impact of employee mental health on innovative behavior is positive (Model 7: β = 0.335; *p* < 0.001). The results indicated that both H3a and H3b were verified by the samples.

Model 4 was built based on model 2, which was applied to test the mediating role of work engagement. The results of model 4 indicated that the coefficient for work engagement is significant (model 4, β = 0.447; *p* < 0.001). However, the coefficient for employee mental health was not significant (model 4, β = 0.129; *ns*). From the results of model 2, model 4 and model 6, we could see that the positive mediating effect of work engagement on the relationship between employee mental health and job performance is significant. Therefore, hypothesis 4a is supported by the samples.

Model 5 was built based on model 2, which was applied to test the mediating role of innovative behavior. The results of model 5 indicated that the coefficient for innovative behavior was significant (model 5, β = 0.444; *p* < 0.001). However, the coefficient for employee mental health is also not significant (model 5, β = 0.108; *ns*). From the results of model 2, model 5 and model 7, we could see that the positive mediating effect of innovative behavior is significant. Therefore, hypothesis 4b is supported by the samples.

The endogeneity problem may be driven by some unobservable characteristics of the firm and employee [69]. Therefore, we consider several control variables in the model. We control the firm age, the number of firm employees, the educational background of employees, and the work experience of employees. Moreover, job satisfaction is considered as a proxy variable of employee mental health. We conduct the hierarchical linear analysis (HLA) and find that Hypotheses 1–3 are supported by data. Therefore, the results show there is not a significant endogeneity problem.

## 5. Discussion

The present study pursued three goals in extending the extant knowledge on the relationship between employee mental health and job performance. First, we set out to investigate how employee’ mental health influences job performance in an emerging economy context. In line with previous theories and research in developed economies [25], we predicted and found that employee mental health also exerts a positive influence on job performance in China. This finding indicates that the mental health of employees is an important factor to predict job performance. Moreover, the result that employee mental health positively affects job performance is robust and valid.

Second, to enrich our insights into the antecedents of job performance, we predicted and found that employee innovation behavior and work engagement positively affect job performance. It is plausible that job performance is enhanced because employees who are more dedicated to work and exhibit more innovative behavior are more effective in meeting the demands of firms, thereby leads to better development of the firm. Importantly, this result extends the findings on factors that promote job performance.

Third, we found that employee mental health is indirectly associated with job performance via innovative behavior and work engagement, which addresses tasks associated with work effectiveness. The positive affect state inherent to mental health is conveyed through innovative behavior and work engagement that are important for work demands. In turn, these two behaviors are positively associated with job performance. These results concerning indirect effects suggest an important nomological chain that begins with the mental health of the employee, which is positively connected with investing more energy and resources via innovative behavior and work engagement to create positive, productive work conditions, leading to better job performance. This indirect connection leads to useful insights into the mental health–job performance relationship.

### 5.1. Theoretical Implications

There are several theoretical implications. Firstly, this study explores the relationship between employee mental health and job performance and tests it in a Chinese context. The important role of employees’ mental health in an organization is a concern for existing scholars. More and more studies observe that mental health strongly influences the individual performance and organizational performance. However, previous studies examine the mental health-job performance relationship in developed countries. Few studies test the relationship in emerging economies such as China. Using the samples from China, we find a positive relationship. By doing so, the findings of this study contribute to the literature on the roles of employees’ mental health in an emerging economy.

Secondly, we identify two types of employee behaviors and analyze their impacts on job performance. Work engagement and innovative behavior are important behaviors that may influence individual work efficiency. In this study, we explore the impacts of work engagement and innovative behavior on job performance. By doing so, the findings extend the antecedents of job performance.

Thirdly, this study reveals the paths of how employee mental health affects job performance, and find that employees’ work engagement and innovative behavior play a positive mediating role in the relationship between employee mental health and job performance. Existing studies have ignored the impact mechanism of employee mental health on job performance. What remains unknown is how employee mental health influences individual performance. Based on the framework of individual characteristics–behaviors–outcome, we view employees’ work engagement and innovative behavior as two salient types of behavior that are affected by mental health. This study examines and finds that employees’ work engagement and innovative behavior mediate the mental health–job performance relationship. These findings further extend the relationship between employee mental health and job performance.

### 5.2. Managerial Implications

The managerial implications of this study are reflected in the following aspects. Firstly, employees (or managers) should pay attention to mental health problem and make necessary adjustments. Poor mental health may lead to absenteeism and low productivity [2]. For employees in a highly competitive environment, the mental health problem is becoming a big challenge for them to improve job performance. Especially under the context of the outbreak of COVID-19, many employees are suffering from mental health problems. It is important for them to maintain their mental health. This requires them to adjust their mental state. Secondly, top managers or leaders in different organizFations could stimulate employees’ work engagement and encourage them to engage in innovative behavior. Especially for employees with good mental health, their intention to work and innovation is strong. How to utilize their passion is important for organization. Hence, managers should observe employees and apply their healthy mentality to improve their job performance. Thirdly, we suggest companies (or organizations) care about employees’ mental health and foster an atmosphere to improve it. Employees are the core capital of companies. Employees with good mental health support enterprises to achieve high organizational performance. Therefore, companies should be concerned about their employees’ mental health and foster a healthy mentality for them.

### 5.3. Limitations and Future Research

There are still some limitations which should be taken into account in future studies. Firstly, the data and samples of this study were collected in China. There are many other emerging economies, such as India and Brazil. In the future, more samples from other emerging economies could be considered to test the model. Additionally, future studies could compare the results of different countries or regions. Secondly, we identify work engagement and innovative behaviors as the mediating variables of the impact of employee mental health on job performance. Are there any other more important mediating variables to be considered? This question should be addressed by future research. Thirdly, we do not consider contextual factor(s) in this study. The relationship between employee mental health and job performance may be influenced by some contextual factors, such as perceived environment, individual personality, and so on. Therefore, future studies could identify certain moderating variables to further explore the relationship between employee mental health and job performance. Finally, this study does not consider the impact of different industries. In different industries, the situation of employees’ mental health may be different, such as the IT industry, manufacturing industry, etc. Hence, future studies could consider the results of different industries, as well as test the model in them.

## 6. Conclusions

Based on existing research gaps, this study explores the impact of employee mental health on job performance. Considering that few studies in the literature focus on the context of China, an emerging economy, this study uses samples in China and finds that employee mental health plays an important role in improving job performance. Chinese organizations should try to maintain employee mental health to achieve high job performance. Furthermore, we discuss the employee mental health–job performance relationship. The paths of how employee mental health influence job performance are revealed in this study. The results show that work engagement and innovative behaviors play positive mediating roles in the relationship between employee mental health and job performance. These findings reveal the relationship between employee mental health and job performance and further enrich the literature on employee behaviors. Furthermore, this study extends the research of employees’ characteristics and their influences on job performance.

## Figures and Tables

**Table 1:** The results of the correlation matrix and descriptive statistics.

	1	2	3	4	5	6	7	Mean	S.D.
Firm age	1							2.978	1.064
Number of employees	0.676 ***	1						3.830	1.420
Education background	0.162 *	0.359 ***	1					2.760	0.790
Work experience	0.202 *	0.186 *	−0.074	1				5.800	4.214
Employee mental health	−0,015	−0.041	−0.056	0.135	1			4.657	1.458
work engagement	0,060	−0.11	−0.073	0.095	0.235 ***	1		4.873	1.288
innovative behavior	−0.036	−0.122	−0.042	0.069	0.382 ***	0.572 ***	1	5.188	1.210
job performance	0.027	−0.031	−0.049	0.162	0.292 ***	0.524 ***	0.609 ***	5.219	1.170

Note: *** *p* < 0.001; * *p* < 0.05.

**Table 2:** The results of regression analysis (models 1–3).

Variables	Dependent Variable: Job Performance		
	Model 1	Model 2	Model 3
Control variables			
Firm age	0.078	0.080	−0.008
Number of employees	−0.070	−0.045	0.019
Education background	0.004	0.032	−0.003
Work experience	0.164	0.126	0.113
Independent variable			
Employee mental health		0.256 **	
Mediating variables			
Work engagement			0.309 **
Innovative behavior			0.295 ***
*R* ^2^	0.031	0.094	0.313
Adj-*R*^2^	0.001	0.058	0.280
F-value	1.038	2.642 *	9.628 ***

Note: *** *p* < 0.001; ** *p* < 0.01; * *p* < 0.05.

**Table 3:** The results of regression analysis (models 4–7).

Variables	Job Performance		Work Engagement	Innovative Behavior
	Model 4	Model 5	Model 6	Model 7
Control variables				
Firm age	0.008	0.023	0.161	0.129
Number of employees	−0.035	0.051	−0.023	−0.217
Education background	0.034	−0.012	−0.005	0.098
Work experience	0.113	0.105	0.030	0.048
Independent variable				
Employee mental health	0.129	0.108	0.284 ***	0.335 ***
Mediating variables				
Work engagement	0.447 ***			
Innovative behavior		0.444 ***		
*R* ^2^	0.273	0.262	0.103	0.142
Adj-*R*^2^	0.239	0.227	0.068	0.109
F-value	7.949 ***	7.524 ***	2.949 **	4.252 ***

Note: *** *p* < 0.001; ** *p* < 0.01.

## Data Availability

The data presented in this study are available upon request from the corresponding author.
